# Canine Cerebrospinal Fluid Analysis Using Two New Automated Techniques: The Sysmex XN-V Body Fluid Mode and an Artificial-Intelligence-Based Algorithm

**DOI:** 10.3390/ani14111655

**Published:** 2024-05-31

**Authors:** Sandra Lapsina, Barbara Riond, Regina Hofmann-Lehmann, Martina Stirn

**Affiliations:** 1 Clinical Laboratory, Department of Clinical Diagnostics and Services, Vetsuisse Faculty, University of Zurich, Winterthurerstrasse 260, CH-8057 Zurich, Switzerland; sandralapsina@live.com (S.L.); rhofmann@vetclinics.uzh.ch (R.H.-L.);; 2 SIA Laboklin, Fridriha Candera iela 4, LV-1046 Riga, Latvia; 3 Roche Pharma Research and Early Development, Pharmaceutical Sciences, Roche Innovation Center, 4070 Basel, Switzerland

**Keywords:** canine, cerebrospinal fluid, Sysmex, body fluid mode, regating, total nucleated cell count, differential count, deep learning, artificial intelligence

## Abstract

**Simple Summary:**

Cerebrospinal fluid analysis is an important diagnostic test for a neurological canine patient. For this analysis, the total nucleated cell count and differential cell counts are routinely taken, but both involve time-consuming manual methods. The Sysmex XN-V body fluid mode is a specific setting used for analyzing different body fluids, including cerebrospinal fluid samples, while the deep-learning-based algorithm generated by the Olympus VS200 slide scanner allows recognition and classification of various cell types on scanned slides. In this study, the faster automated methods mentioned above were assessed by comparing them with the manual methods for both the total nucleated and differential cell counts. Manually set gates were used for the Sysmex body fluid mode following incorrect cell classifications recorded when using the predefined settings. This study demonstrates that automated methods may be used for total nucleated cell count assessments in canine cerebrospinal fluid samples, but optimization is still needed for both differential counts.

**Abstract:**

Cerebrospinal fluid analysis is an important diagnostic test when assessing a neurological canine patient. For this analysis, the total nucleated cell count and differential cell counts are routinely taken, but both involve time-consuming manual methods. To investigate faster automated methods, in this study, the Sysmex XN-V body fluid mode and the deep-learning-based algorithm generated by the Olympus VS200 slide scanner were compared with the manual methods in 161 canine cerebrospinal fluid samples for the total nucleated cell count and in 65 samples with pleocytosis for the differential counts. Following incorrect gating by the Sysmex body fluid mode, all samples were reanalyzed with manually set gates. The Sysmex body fluid mode then showed a mean bias of 15.19 cells/μL for the total nucleated cell count and mean biases of 4.95% and −4.95% for the two-part differential cell count, while the deep-learning-based algorithm showed mean biases of −7.25%, −0.03% and 7.27% for the lymphocytes, neutrophils and monocytoid cells, respectively. Based on our findings, we propose that the automated Sysmex body fluid mode be used to measure the total nucleated cell count in canine cerebrospinal fluid samples after making adjustments to the predefined settings from the manufacturer. However, the two-part differential count of the Sysmex body fluid mode and the deep-learning-based algorithm require some optimization.

## 1. Introduction

The analysis of cerebrospinal fluid (CSF) serves as a crucial diagnostic test when assessing patients with neurological diseases. The analysis is sensitive but unspecific and can provide valuable information when evaluating central and peripheral neurological diseases with infectious, inflammatory, traumatic, degenerative and neoplastic causes [1].

Routine CSF assessment includes both qualitative macroscopic evaluation of the sample and quantitative measurements of the total nucleated cell count (TNCC), differential cell count, erythrocyte count and total protein concentration.

In healthy canine patients, the TNCC is very low (reference interval up to 5 cells/µL) for samples collected from either the cerebromedullary or lumbar cistern [2,3,4]. An increased TNCC is called pleocytosis, the gradation of which—as mild (6–50 cells/µL), moderate (51–500 cells/µL) or marked (>500 or 1000 cells/µL)—is rather subjective [5,6]. The manual method of using a hemacytometer (either a Fuchs-Rosenthal or Neubauer counting chamber) has long been viewed as the “gold standard” for TNCC measurements in the CSF since the cellularity is considered too low to be measured with a hematology analyzer [1].

A differential cell count of 100 cells is usually taken after cells have been concentrated by either centrifugation, sedimentation or membrane filtration [1,5]. The relative numbers of monocytoid cells, lymphocytes, neutrophils and eosinophils are obtained manually by counting via light microscopy [1].

However, manual counting methods are time-consuming, labor-intensive and repetitive, require experienced staff and can lead to considerable inter- and intra-observer variability [7,8]. Therefore, automated methods for cell quantification and differentiation in CSFs are being explored.

In the last 25 years, a number of studies have assessed automated TNCC and differential cell count measurements in the CSF using either impedance or flow cytometry methods [7,9,10,11,12,13,14,15,16,17,18,19,20,21]. In addition to the routinely available blood mode on every hematology analyzer, in 2005, Siemens introduced a specific CSF analysis mode on their ADVIA analyzer [19], while, in 2007, Sysmex introduced a body fluid (BF) mode on their XE series [22]. The Sysmex BF offers measurements of both the TNCC (TC-BF; in this text, referred to as BF-TNCC) and white blood cell count (WBC) (WBC-BF; in this text, referred to as BF-WBC). The difference between these two parameters is that the BF-WBC recognizes and counts only leukocytes, while the BF-TNCC also counts large and highly fluorescent cells such as mesothelial cells [23]. Additionally, a two-part differentiation into mononuclear (MN) and polymorphonuclear (PMN) cells is also performed using flow cytometry. Furthermore, it is possible to develop specific profiles by setting manual gates on the analyzer in the Sysmex BF mode. The two main advantages provided by the BF mode are its automated washing step with a subsequent background check whenever the BF mode is accessed and its capacity to count three times as many cells per measurement when compared with the blood mode [24].

In veterinary medicine, only a few studies on automated CSF measurements are available, all of them in dogs and obtained using hematology analyzers operating based on flow cytometry. In terms of assessing specific BF modes, two studies were undertaken that tested the ADVIA 2120 CSF analysis mode, and one study evaluated the Sysmex BF mode. A correlation of r > 0.85 was noted for the TNCC in all three studies when excluding outliers [7,19,25]. However, for the two-part differentiation into MN and PMN cells, their results varied notably: the ADVIA 2120 CSF analysis mode showed a correlation of r = 0.86 for both MN and PMN cells, while the Sysmex XN BF mode showed a nonexistent correlation of r = <0.03 for the two cell populations [19,25]. For differentiation into individual cell populations, the correlation seemed to depend on cellularity. The ADVIA 2120 CSF analysis mode demonstrated a correlation of r = 0.92 for lymphocytes, r = 0.77 for neutrophils and r = 0.70 for monocytoid cells in samples with pleocytosis, while in unremarkable CSF samples, the correlation was only r < 0.53 and thus notably lower than those for all three cell populations [7,19].

Another rapidly developing area with huge potential is artificial intelligence (AI), particularly its subdivision of machine learning, which can make predictions and decisions similarly to the human mind. One subset of machine learning, deep learning, requires remarkably low human intervention since it automatically extracts the identifying features based on which it builds algorithms of artificial neural networks. Deep learning is being progressively applied in digital cytology since it allows for the quick processing of large amounts of data and does not require staff with specific information technology knowledge. Recent advancements in AI, particularly machine learning and deep learning, have shown promise in identifying cellular abnormalities in various medical contexts. For example, a 2021 study demonstrated 95% accuracy in recognizing specific cell types in human CSF samples using a deep-learning-based algorithm. The deep learning algorithm also showed an 86% reduction in the turnaround time when compared to the manual technique [26]. In a similar study with a five-part differential count (lymphocytes, monocytoid cells, neutrophils, erythrocytes and cancer cells), the deep-learning-based algorithm demonstrated a predictive average precision of 91.1% to 98.7%, thus accurately recognizing even cancer cells and outperforming the manual differentiation by interns and junior doctors while showing close results to the differentiation performed by experts [27].

Based on the extant literature and a similar study that has been recently published by the authors on equine bronchoalveolar lavage samples [28], we hypothesized that it is possible to obtain accurate automated measurements for both the TNCC and the differential cell count in CSF samples. We also hypothesized that the automated methods are more precise than the manual methods due to the higher numbers of cells counted. Accordingly, the aims of this study were as follows:

1. To compare the Sysmex XN-V BF mode with manual techniques for taking the TNCC and the two-part differential cell count.
2. To compare the Olympus VS200 software’s deep-learning-based algorithm with manual techniques for taking the three-part differential cell count on digital images of scanned CSF cytospin preparations.

## 2. Materials and Methods

### 2.1. Study Design

This prospective study was conducted between July 2020 and October 2022 in the Clinical Laboratory of the Vetsuisse Faculty, University of Zurich (Switzerland) according to the Swiss law. All analyses were performed using leftover material from fresh daily routine diagnostic samples. No additional samples or volumes were collected for this study. Samples were submitted for routine diagnostic purposes by veterinarians from the Clinic for Small Animal Medicine, University Animal Hospital Zurich of the Vetsuisse Faculty, University of Zurich. All samples were collected by the attending veterinarian in plain tubes. The TNCC was taken and cytospin preparations were assembled within two hours after sampling, and the cytospin preparations were evaluated via light microscopy on the same day. Later, the archived cytospin preparations were scanned, and the digital images were assessed via AI, for all slides at once. For the comparison study (Table 1), the TNCC and the two-part differential cell count as obtained with the Sysmex XN-V (Sysmex Corporation, Kobe, Japan) BF mode were compared with the results from the manual techniques. To improve the experiment, the gating of the scattergrams produced by the Sysmex XN-V BF mode was manually optimized for the canine CSF samples, and all samples of the comparison study were then reanalyzed using the new gates (see the Results section). Next, our cytospin preparation slides were scanned and processed with the Olympus Slideview VS200 slide scanner (Olympus Corporation, Hachioji, Tokyo, Japan), and afterward, three-part differential cell counts of the digital images were taken both manually and using the VS200 software (version 3.3 (Olympus Soft Image Solution GmbH, Münster, Germany) with an AI neural network algorithm (which was developed via training before beginning the comparison study); moreover, the slides were also manually differentiated via light microscopy (Table 1). In total, 161 routine CSF samples from dogs presenting for either a clinical workup or follow-up due to various neurological disorders were included in the comparison study. The manual TNCC was taken by experienced laboratory technicians, while all manual differential cell counts both via light microscopy and on digital images, as well as the training of the AI neural network algorithm, were performed by one of the authors, a senior resident of veterinary clinical pathology (S.L.). For instrument validation of the Sysmex XN-V BF mode for canine CSF samples, both systematic and random error were assessed by a method comparison study and a precision study for the test and reference method. Furthermore, linearity was tested for the Sysmex XN-V [29].

### 2.2. Training the AI Neural Network Algorithm

Training of the AI neural network algorithm for canine CSF samples was performed as described previously [28]. Shortly, cytospin preparations of 20 arbitrarily chosen routine canine CSF samples with morphologically well-preserved cells when assessed via light microscopy were scanned with the Olympus Slideview VS200 slide scanner (Olympus, Shinjuku, Japan) at 40× magnification (oil immersion) to obtain virtual slides. These were further processed with the VS200 software, taking 40 digital images in the brightfield imaging mode from the monolayer areas with the best cytological quality. All cells on all images were manually labeled as either monocytoid cells, lymphocytes or neutrophils. The total cell count on each image ranged between 26 and 88 cells, in total reaching 2240 cells on all digital images, and the cell-type distribution was as follows: 529 monocytoid cells, 832 lymphocytes and 879 neutrophils. To establish a neural network algorithm, several configurations offered by the VS200 software were applied. The maximum similarity of 0.75 was reached with multi-label classification and a specific network (RGB) after 1,000,000 iterations. Increasing the number of training images did not further improve the similarity at this point, and so we considered the AI neural network algorithm to be trained and ready for further use in this study.

### 2.3. TNCC

The TNCC obtained with the automated hematology analyzer Sysmex XN1000-V (Sysmex Corporation, Kobe, Japan) using software version 3.04-00 in the BF mode was compared with the manual TNCC obtained with a hemacytometer (Table 1). The automated measurements with the Sysmex XN-V and manual TNCC were taken from the same aliquot tube, which was thoroughly mixed on the nutating laboratory tube mixer (VWR International, Radnor, PA, USA) for five minutes. The TNCC was graded as follows: unremarkable, 0–5 cells/µL; mild pleocytosis, 6–50 cells/µL; moderate pleocytosis, 51–500 cells/µL; and marked pleocytosis, >500 cells/µL. This grading of pleocytosis is subjective and, to the authors’ knowledge, the applied grading protocol is partly based on a study conducted on cats in 2005 [6].

#### 2.3.1. Manual TNCC

The manual TNCC was taken as follows: a Fuchs-Rosenthal hemocytometer (Hecht Assistant, Altnau, Germany) was filled with 90 µL of the CSF mixed with 10 µL of Samson’s concentrate (Artechemis, Zofingen, Switzerland) and left to sediment for five minutes. Afterward, all nucleated cells were counted in the chamber’s 16 large squares on both sides, and the average number of cells per side was determined and then divided by three to obtain the number of cells per µL (cells/µL).

#### 2.3.2. Automated TNCC Count with Sysmex XN-V

The Sysmex XN-V uses fluorescence flow cytometry, and the results are reported as cells/µL. Quality control (QC) was performed daily before processing routine samples using the XN-CHECK 3 Level controls (Sysmex Corporation, Kobe, Japan), covering both the normal range and abnormally low and high tested parameters. Additionally, a background check was performed every time BF analysis was conducted, with an acceptable BF WBC value of 0.001 × 10^3^ cells/µL or less [30,31].

### 2.4. Two-Part Differential Cell Count

The two-part differential count was taken only in samples with pleocytosis (TNCC > 5 cells/µL), classified as such by at least one of the TNCC methods, which amounted to 65 samples of the total 161 samples of this study. The automated two-part differential count in the Sysmex BF mode was compared with the manual two-part 100-cell differential count obtained via light microscopy (Table 1). The samples were further classified by the type of pleocytosis: >70% MN% as mononuclear pleocytosis, >70% PMN% as polymorphonuclear pleocytosis and other samples as mixed-cell pleocytosis.

#### 2.4.1. Manual Two-Part Differential Cell Count

The cytospin preparations were assembled as follows: First, 0.5 mL of the CSF (from the same aliquot tube that the Sysmex BF mode automated measurements were performed on) was spun in 406 g for five minutes to obtain a cell pellet. The supernatant was then removed, leaving approximately 0.1 mL of fluid with the pellet, and then 0.4 mL of laboratory-prepared 5% bovine serum albumin solution (Sigma-Aldrich, St. Louis, MO, USA) was added to the tube to return its contents to the initial total fluid volume of 0.5 mL. Following that, the tube contents were thoroughly mixed with a pipette five times. Three drops of the obtained fluid were then spun at 72× *g* for 10 min using the cytocentrifuge Shandon Cytospin 4 (Thermo Fisher Scientific, Waltham, MA, USA). The cytospin preparation slides were then air-dried and stained with the modified Wright–Giemsa stain from the Hematek Stain Pak (Siemens, Munich, Germany) on a Hematek 4488C slide stainer (Siemens, Munich, Germany). Both the cytospin preparation and the staining process were highly standardized.

The differential count was taken via brightfield light microscopy on an Olympus BX53 microscope (Olympus, Shinjuku, Japan) at a 500× magnification. First, a manual four-part 100-cell differential cell count of monocytoid cells, lymphocytes, neutrophils and eosinophils was obtained from an area of well-dispersed cells. Then, the percentages of monocytoid cells and lymphocytes were summed to obtain the MN%, while the percentages of neutrophils and eosinophils were summed similarly to obtain the PMN%.

#### 2.4.2. Automated Sysmex BF Two-Part Differential Cell Count

The Sysmex XN-V BF mode offers a two-part differential count in MN or PMN cells using fluorescence flow cytometry. The samples were thoroughly mixed as described above for the automated TNCC. The results were reported as percentages for the relative cell counts.

### 2.5. Three-Part Differential Cell Count

The three-part 100-cell differential cell count was taken only in samples with pleocytosis (TNCC > 5 cells/µL), classified as such by at least one of the TNCC methods. The cytospin preparations were scanned with the Olympus Slideview VS200 slide scanner and processed with the VS200 software using the herein-developed AI neural network algorithm. Digital images were taken of approximately 100 cells from each sample. Those images were taken from well-dispersed areas, where the cells were located individually and touched as little as possible. The three-part 100-cell differential cell count of monocytoid cells, lymphocytes and neutrophils was then performed on these digital images. Each image was counted both manually and with the pre-developed AI algorithm, and then the two methods were compared. In addition, a three-part differential cell count was also taken via light microscopy for each sample on the cytospin preparations. The samples were then classified by the type of pleocytosis, that is, as having either lymphocytic (≥70% lymphocytes), mononuclear (≥70% mononuclear cells, but <70% lymphocytes), mixed-cell (all cell types <70% and mononuclear pleocytosis does not apply) or neutrophilic (≥70% neutrophils) pleocytosis [5].

### 2.6. Precision and Linearity

With all counting methods performed, we tested the precision for both the TNCC and the differential cell count. The precision for the TNCC and the two-part differential count was assessed in samples with low and high cell counts with both manual methods and the Sysmex XN-V analyzer in the BF mode. The precision for the three-part differential cell count was assessed in three CSF samples by performing a 100-differential cell count in well-dispersed areas with the following three methods: manually via light microscopy on cytospin preparations, manually on digital images and with the AI algorithm on digital images. To test the AI precision with more counted cells, an 800-cell three-part differential cell count was taken on digital images of the same three samples using the AI algorithm. All precision experiments were performed by repeating the measurements 6–10 times within a run, using a subset of both the 161 samples from the comparison study and other routine samples not part of the comparative study.

The linearity of the TNCC obtained with the Sysmex BF was measured within-run with five dilutions (20%, 40%, 60%, 80%, 100%) from a routine sample (BF-TNCC of 199 cells/µL).

### 2.7. Statistical Analysis

Passing–Bablok regression and a Bland–Altman difference plot were used to compare the methods and assess the bias [32]. Spearman’s rank correlation coefficient (r) was used to determine the correlation between different methods [33]. To assess precision, the standard deviation (SD) and coefficient of variation (CV) were calculated. Linear regression was applied to evaluate linearity. Wilcoxon’s signed-rank test was used to compare the measurements of the same parameter in the same sample, considering a *p*-value < 0.05 as statistically significant. The statistical analysis was performed with Analyse-it on Microsoft Excel version 2108 (Build 14326.20404).

## 3. Results

### 3.1. Sysmex XN-V BF Mode Versus Manual Methods: TNCC

**Figure 1:**
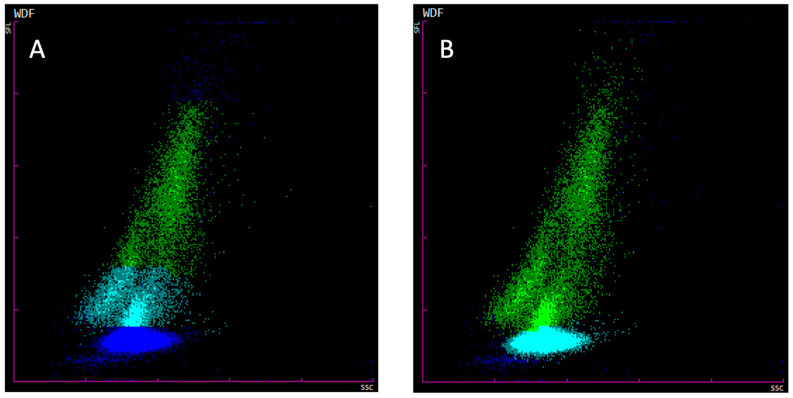
Comparison of representative Sysmex XN-V BF mode scattergrams before (**A**) and after (**B**) setting of manual gates. Debris is depicted as dark blue, MN cells are green, and PMN cells are light blue. Before manual regating (**A**), most of the PMN were are classified as debris, while MN cells were partly counted as debris, MN cells and PMN cells. After manual regating (**B**), the cell populations were correctly distinguished.

The TNCC was obtained using the Sysmex XN-V BF mode and manual methods. The former allows for the classification of events into debris, MN cells and PMN cells by separating these entities into different colors. When analyzed by the Sysmex BF mode, an incorrect automated gating for both MN and PMN cells was observed on the scattergrams of many CSF samples (for an example, see Figure 1A; after manual regating, see Figure 1B). The issue manifested in frequent misidentification of distinctly demarcated cell populations on the scattergram, classifying the PMN cells as debris while the MN cells were depicted as a mixture of debris, MN and PMN cells. To solve this issue, before including the samples in the comparison study, a manual gate was established in the extended scattergram of one of the CSF samples with clearly identifiable PMN and MN cell populations. This manual gate was then further applied to all other samples while carefully assessing its suitability in each case. The established manual gate fit all samples according to visual inspection of the scattergrams. In this text, only the results obtained after regating are reported.

**Figure 2:**
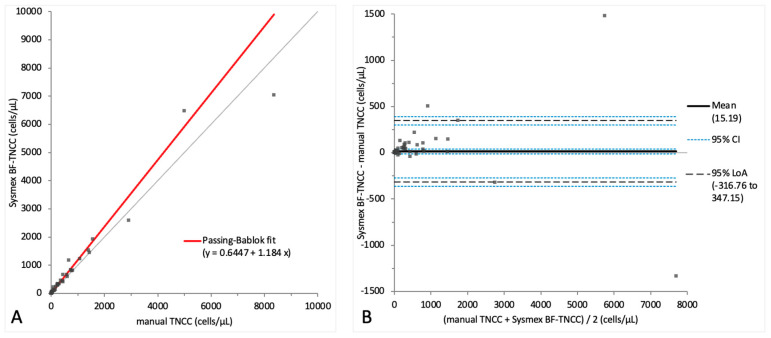
Agreement between the manual TNCC and Sysmex BF-TNCC (cells/µL). The graph on the left (**A**) is a Passing–Bablok regression analysis with intercept 0.64 (from 0.25 to 0.66) and slope 1.18 (from 1.14 to 1.27). The graph on the right (**B**) is a Bland–Altman difference plot. The thin horizontal gray line (0 at the *y*-axis) is the line of identity, and the thick black line indicates the bias (mean difference between methods), with its confidence intervals as thin blue dashed lines. The black dashed horizontal lines are the 95% limits of agreement with their 95% confidence intervals as the thin blue dashed lines. The mean difference is 15.19 (from −11.17 to 41.56) cells/µL, the lower limit of agreement is −316.76 (from −361.91 to −271.62) cells/µL and the upper limit of agreement is 347.15 (from 302.01 to 392.30) cells/µL. Numbers in parentheses are 95% confidence intervals.

Comparison results for the Sysmex BF mode and the manual TNCC measurements are depicted in Table 2 and Figure 2. The BF-TNCC and the BF-WBC yielded identical values without any statistically significant difference; therefore, only the BF-TNCC is further reported herein (Figure 2A,B). Our comparison of the Sysmex BF-TNCC with the manually obtained TNCC revealed a positive proportional systematic bias in the Passing–Bablok regression analysis (Figure 2A). The Bland–Altman difference plot, meanwhile, showed a small mean bias with moderately wide limits of agreement indicating random error (Figure 2B). A correlation of r = 0.91 was observed between the BF-TNCC and the manual TNCC, and we found a statistically significant difference between the TNCC measurements of both methods (*p*-value < 0.001). The raw data for the TNCC measurements are depicted in Table S1.

With the TNCC cut-off set at ≤5 cells/µL for unremarkable CSF samples [2,3,4], 65/161 (40.4%) samples were classified as abnormal by the BF-TNCC and 56/161 (34.8%) by the manual TNCC (Table 3). Thus, the Sysmex BF-TNCC classified nine more samples as having pleocytosis than the manual TNCC. When inspecting these, we found that eight of the samples showed borderline TNCC in the range of 4–6 cells/µL, thus being close to classification cut-off. All samples classified as abnormal in the manual TNCC were also classified as such in the BF-TNCC (Table S1).

### 3.2. Sysmex XN-V BF Mode Versus Manual Methods: Two-Part Differential Cell Count

**Figure 3:**
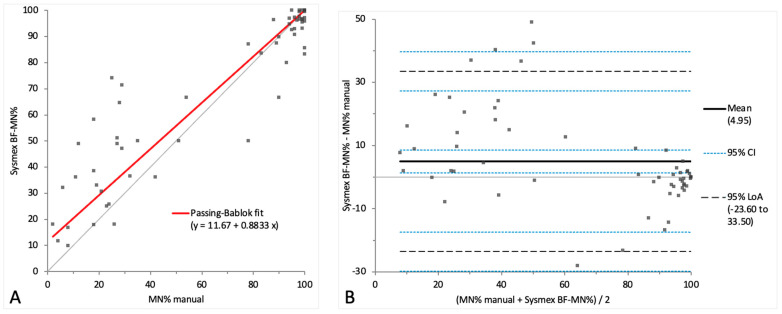
Agreement between the manual MN% and Sysmex BF-MN%. The graph on the left (**A**) is a Passing–Bablok regression analysis with intercept 11.67 (from 2.99 to 23.08) and slope 0.88 (from 0.77 to 0.97). The graph on the right (**B**) is a Bland–Altman difference plot. The thin horizontal gray line (0 at the *y*-axis) is the line of identity, and the thick black line indicates the bias (mean difference between methods), with its confidence intervals as thin blue dashed lines. The black dashed horizontal lines are the 95% limits of agreement with their 95% confidence intervals as the thin blue dashed lines. The mean difference is 4.95 (from 1.34 to 8.56) %, the lower limit of agreement is −23.60 (from −29.80 to −17.40) % and the upper limit of agreement is 33.50 (from 27.30 to 39.70) %. Numbers in parentheses are 95% confidence intervals.

**Figure 4:**
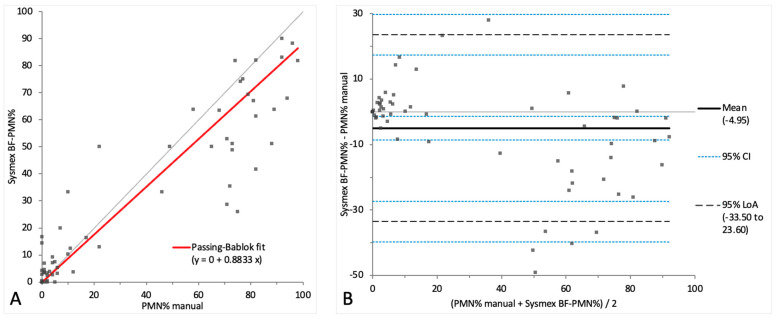
Agreement between the manual PMN% and Sysmex BF-PMN%. The graph on the left (**A**) is a Passing–Bablok regression analysis with intercept 0 (from 0 to 1.69) and slope 0.88 (from 0.77 to 0.96). The graph on the right (**B**) is a Bland–Altman difference plot. The thin horizontal gray line (0 at the *y*-axis) is the line of identity, and the thick black line indicates the bias (mean difference between methods), with its confidence intervals as thin blue dashed lines. The black dashed horizontal lines are the 95% limits of agreement with their 95% confidence intervals as the thin blue dashed lines. The mean difference is −4.95 (from −8.56 to −1.34) %, the lower limit of agreement is −33.50 (from −39.70 to −27.30) % and the upper limit of agreement is 23.60 (from 17.40 to 29.80) %. Numbers in parentheses are 95% confidence intervals.

To compare the Sysmex BF mode with the manual method for the two-part differential cell count in samples showing pleocytosis, the BF-MN% was compared with the combined percentage of MN cells (monocytoid cells and lymphocytes) from the manual four-part 100-cell differential count (Figure 3A,B). In addition, the BF-PMN% was compared with the manually obtained combined percentage of PMN cells (neutrophils and eosinophils) (Figure 4A,B). The comparison results of the automated and manual methods for the two-part differential in samples with pleocytosis are depicted in Table 2. The raw data for the two-part differential cell count are depicted in Table S2.

A proportional systemic bias was noted in the Passing–Bablok regression analysis for both MN% and PMN% (Figure 3A and Figure 4A), while a small mean bias with moderate limits of agreement was present on the Bland–Altman difference plot, indicating random error (Figure 3B and Figure 4B). A correlation of r = 0.89 was found between the methods’ measurements for both MN% and PMN%, with a statistically significant difference (*p*-value 0.03).

All samples with pleocytosis were classified by their type, according to the measurements obtained both by the Sysmex BF mode and manually via light microscopy. When classifying the samples by the type of pleocytosis, according to the results obtained by the Sysmex BF, 39/65 (60%) of all samples met the criteria for mononuclear, 18/65 (27.7%) for mixed-cell and 8/65 (12.3%) for polymorphonuclear pleocytosis. Meanwhile, in the results obtained manually via light microscopy, 39/65 (60.0%) of all samples were classified as having mononuclear, 6/65 (9.2%) mixed-cell and 20/65 (30.8%) polymorphonuclear pleocytosis (Table 4). Of the 65 samples showing pleocytosis, 51 had concordant classifications for the type of pleocytosis. Of the aforementioned samples, 37 were classified as having mononuclear, 8 polymorphonuclear and 6 mixed-cell pleocytosis. A further 14 samples showed discrepant pleocytosis classifications between different pleocytosis groups, though 9 of those samples could be considered borderline, lacking a difference of greater than 5% past the border value in either MN% or PMN% for classification in a different pleocytosis category (Table S2).

### 3.3. Olympus VS200 Slide Scanner and Software-Generated Deep-Learning-Based Algorithm Versus Manual Methods: Three-Part Differential Cell Count

**Figure 5:**
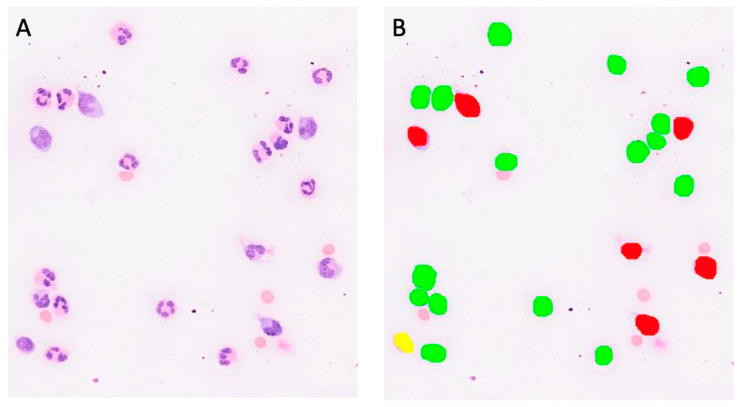
Digital images of the same location of a virtual cytospin preparation slide in the brightfield imaging mode. (**A**)–native image; (**B**)–after being analyzed with a deep learning neural network. Color code: red–monocytoid cells; yellow–lymphocytes; green–neutrophils.

Using the Olympus Slideview VS200 slide scanner in the brightfield imaging mode, between one and six images were taken from each virtual cytospin preparation slide of all 65 samples with pleocytosis, to give a total number of approximately 100 cells per virtual slide. Two of the cytospin preparation slides were damaged while wiping off oil after the light microscopy, and another slide was so cellular that both staining and visual recognition of individual cells were severely impaired on most of the cytospin preparation area; these three samples were therefore excluded from this part of the study. From the remaining 62/65 virtual cytospin preparation slides, a total of 120 digital images was obtained in well-dispersed monolayer areas. Each image was counted both manually and with a pre-developed AI algorithm, allowing us to obtain a differential count in three categories: monocytoid cells, lymphocytes and neutrophils (Figure 5). One of the samples demonstrated eosinophilic pleocytosis, showing 67% eosinophils in the manual four-part 100-cell differential count by light microscopy, and, in contrast, a few other cytospin preparation slides contained very few eosinophils, which never exceeded 3% in the differential cell count via light microscopy. Generally, apart from the one sample with eosinophilic pleocytosis, the number of eosinophils in the other samples was deemed to be negligible in the differential cell count, and since samples with a sufficient number of eosinophils to adequately train the AI algorithm were hard to come by, no specific AI algorithmic training was undertaken for this specific cell population. According to the authors’ visual observation, the eosinophils were misclassified as neutrophils in most cases. The raw data for the three-part differential cell count are depicted in Table S3.

**Figure 6:**
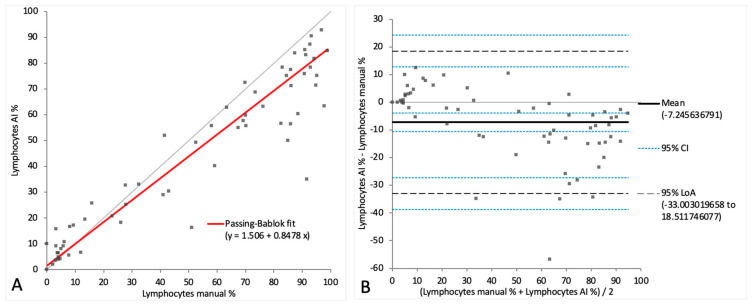
Agreement between manually and artificial intelligence (AI)-algorithm-counted lymphocytes on images of virtual cytospin preparation slides. The graph on the left (**A**) is a Passing–Bablok regression analysis with intercept 1.51 (from −0.03 to 4.99) and slope 0.85 (from 0.79 to 0.90). The graph on the right (**B**) is a Bland–Altman difference plot. The thin horizontal gray line (0 at the *y*-axis) is the line of identity, and the thick black line indicates the bias (mean difference between methods), with its confidence intervals as thin blue dashed lines. The black dashed horizontal lines are the 95% limits of agreement with their 95% confidence intervals as the thin blue dashed lines. The mean difference is −7.25 (from −10.58 to −3.91) %, the lower limit of agreement is −33.00 (from −38.74 to −27.27) % and the upper limit of agreement is 18.51 (from 12.78 to 24.25) %. Numbers in parentheses are 95% confidence intervals.

**Figure 7:**
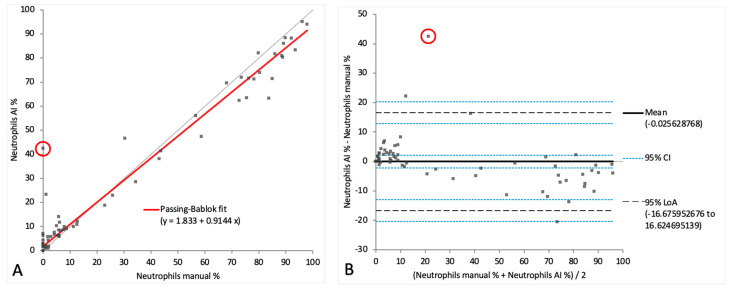
Agreement between manually and artificial intelligence (AI)-algorithm-counted neutrophils on images of virtual cytospin preparation slides. The graph on the left (**A**) is a Passing–Bablok regression analysis with intercept 1.83 (from 0.86 to 2.89) and slope 0.91 (from 0.87 to 0.95). The graph on the right (**B**) is a Bland–Altman difference plot. The thin horizontal gray line (0 at the *y*-axis) is the line of identity, and the thick black line indicates the bias (mean difference between methods), with its confidence intervals as thin blue dashed lines. The black dashed horizontal lines are the 95% limits of agreement with their 95% confidence intervals as the thin blue dashed lines. The mean difference is −0.03 (from −2.18 to 2.13) %, the lower limit of agreement is −16.68 (from −20.38 to −12.97) % and the upper limit of agreement is 16.62 (from 12.92 to 20.33) %. The red circles depict the sample with marked eosinophilic pleocytosis. Numbers in parentheses are 95% confidence intervals.

**Figure 8:**
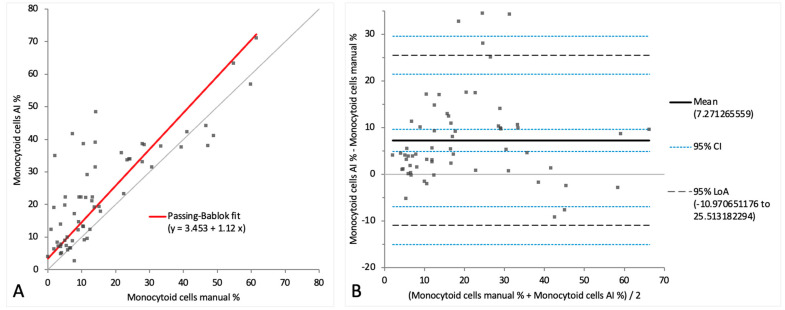
Agreement between manually and artificial intelligence (AI)-algorithm-counted monocytoid cells on images of virtual cytospin preparation slides. The graph on the left (**A**) is a Passing–Bablok regression analysis with intercept 3.45 (from 0.25 to 6.71) and slope 1.12 (from 0.95 to 1.42). The graph on the right (**B**) is a Bland–Altman difference plot. The thin horizontal gray line (0 at the *y*-axis) is the line of identity, and the thick black line indicates the bias (mean difference between methods), with its confidence intervals as thin blue dashed lines. The black dashed horizontal lines are the 95% limits of agreement with their 95% confidence intervals as the thin blue dashed lines. The mean difference is 7.27 (from 4.91 to 9.63) %, the lower limit of agreement is −10.97 (from −15.03 to −6.91) % and the upper limit of agreement is 25.51 (from 21.45 to 29.57) %. Numbers in parentheses are 95% confidence intervals.

When comparing the cell categories between the AI-algorithm-based identification and the manual differentiation on digital images, lymphocytes and neutrophils showed correlations of r = 0.90 and r = 0.91, respectively. Passing–Bablok regression analysis revealed intercepts of between 1.51 and 3.45 and slopes of between 0.85 and 1.12. (Figure 6A and Figure 7A). On the Bland–Altman difference plot, a small mean difference of −7.25% (CI: from −10.58 to −3.91) for lymphocytes and very small mean difference of −0.03% (CI: from −2.18 to 2.13) for neutrophils were observed, with moderately wide limits of agreement indicating a moderate random error (Figure 6B and Figure 7B). The sample with the marked eosinophilic pleocytosis was seen as an outlier in both the Passing–Bablok regression analysis and the Bland–Altman difference plot for neutrophils (Figure 7A,B). The monocytoid cell population showed a correlation of r = 0.78 and a proportional systemic bias in the Passing–Bablok regression analysis (intercept 3.45, slope 1.12) (Figure 8A), while on the Bland–Altman difference plot, a small mean difference of 7.27% (CI: from 4.91 to 9.63) was observed, with moderately wide limits of agreement indicating a moderate random error (Figure 8B). A statistically significant difference between the measurements of the two methods was seen in the case of the neutrophils and the monocytoid cells (*p*-values 0.001 and <0.0001, respectively) but not for the lymphocytes (*p*-value 0.56).

**Figure 9:**
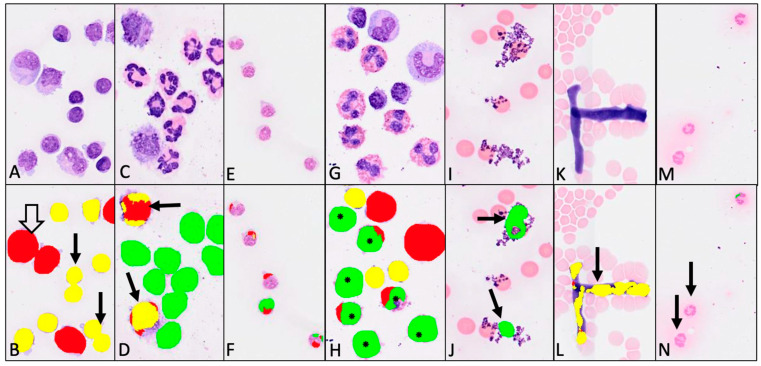
Misclassification issues with artificial intelligence (AI) algorithm. (**A**,**C**,**E**,**G**,**I**,**K**,**M**)—native digital images from different locations on the virtual cytospin preparation slides; (**B**,**D**,**F**,**H**,**J**,**L**,**N**)—corresponding classification performed by the AI algorithm on the same locations. (**A**,**B**)—pair of lymphocytes counted as one (black arrow), and pair of monocytoid cells counted as one (white arrow); (**C**,**D**)—two monocytoid cells fragmentally classified as eight cells, comprising four monocytoid cells and four lymphocytes (arrow); (**E**,**F**)—due to pale staining, all lymphocytes were fragmentally misclassified as monocytes and neutrophils; (**G**,**H**)—eosinophils were misidentified as neutrophils and monocytoid cells (*); (**I**,**J**)—staining artifacts misclassified as neutrophils (arrow); (**K**,**L**)—unidentified artifact counted as several lymphocytes and a monocytoid cell (arrow); and (**M**,**N**)—due to pale staining of three neutrophils, two were not detected (arrow). Color code: red—monocytoid cells, yellow—lymphocytes, green—neutrophils.

Regarding the differences noted between the methods, and also regarding the observed outliers, we detected several misclassification issues with the neural network. For instance, closely located cells in the same category were often counted as one (Figure 9A,B). This issue was most frequently observed in dense regions of the virtual cytospin preparation slides. Alternatively, the recognition of some cells was fragmented, and one cell was thus counted as several cells of either the same or different categories (Figure 9C–H). A further issue was that staining artifacts and unidentified artifacts were often misidentified as various cell categories (Figure 9I–L). Moreover, in the case of paler stained cytospin preparations, the cells were either misidentified as different cell populations (Figure 9E,F) or remained unrecognized (Figure 9M,N). Finally, eosinophils were misclassified as neutrophils in most cases or occasionally as monocytoid cells (Figure 9G,H).

All samples with pleocytosis were classified by their type, according to the three-part differential cell count results provided by each of the following three methods: differential cell count via the AI algorithm on digital images, manual differential cell count on digital images and manual differential cell count via light microscopy on cytospin preparations. According to the AI algorithm on digital images, 15/62 (24.2%) samples were classified as lymphocytic, 10/62 (16.1%) as mononuclear, 20/62 (32.3%) as mixed-cell and 17/62 (27.4%) as neutrophilic pleocytosis, while for the manual differentiation on digital images, the respective results were as follows: 18/62 (29.0%), 7/62 (11.3%), 10/62 (16.1%) and 26/62 (41.9%). The light microscopy provided the following results: 26/62 (41.9%) for lymphocytic, 12/62 (19.4%) for mononuclear, 4/62 (6.5%) for mixed-cell and 19/62 (30.6%) for neutrophilic pleocytosis. The sample with the eosinophilic pleocytosis was classified as such by both manual methods and misclassified as a mixed-cell pleocytosis by the AI algorithm (Table 5). Of all 62 samples classified as having pleocytosis, only 4 received a concordant classification by all three methods, 1 for each category of neutrophilic, lymphocytic, mononuclear and mixed-cell pleocytosis. The greatest concordance between the methods was observed for both methods operating on digital images, with which 45/62 samples (72.6%) were classified into the same category (16 as having neutrophilic, 15 lymphocytic, 8 mixed-cell and 6 mononuclear pleocytosis). The other 17/62 samples (27.4%) were classified in different pleocytosis categories by the three methods. The greatest incidence of pleocytosis misclassification was observed for the manual count via light microscopy on the cytospin preparations, with lymphocytic pleocytosis most often being misclassified as neutrophilic pleocytosis and vice versa (Table S3).

### 3.4. Precision of Different Methods and Linearity of the Sysmex BF Mode

A comparison of the precision of the TNCCs from the Sysmex BF mode and the manual method, for samples with low and high cell counts, is depicted in Table 6. Note that all Sysmex BF measurements assessed were regated ones, due to the previously described cell misclassification issue before regating. The CV for the low cell count as obtained by the Sysmex BF-TNCC was 21.5% and thus notably lower than the CV for the manual method, which was 47.5%. Meanwhile, the CVs for the high cell count were similar for the two methods, at 3.8% for the manual method and 4.8% for the Sysmex BF mode.

**Figure 10:**
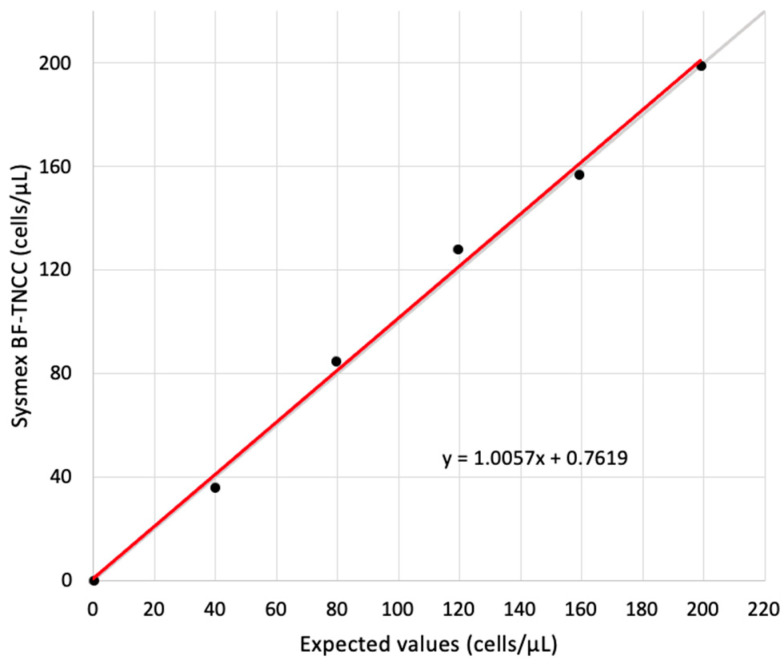
Linearity for Sysmex BF-TNCC depicted as a linear regression. The intercept is 0.76, and the slope is 1.01. The gray line is the identity line.

The linearity for Sysmex BF-TNCC was good and showed a recovery of between 90% and 108% up to a cellularity of 199 cells/µL (Figure 10).

A precision study was performed for the three-part differential cell count with the following three methods: (1) 100-cell differential count manually via light microscopy on cytospin preparations, (2) 100-cell differential count manually on digital images and (3) 100-cell differential count with AI algorithm on digital images. To assess the performance of the AI algorithm when counting more cells, an additional 800-cell differential cell count was performed on digital images with the AI algorithm (Table 7). For this precision study, three samples of good quality and high cellularity were selected, with cell populations that were morphologically well distinguishable via light microscopy. For the 100-cell differential count, the AI algorithm showed the lowest CVs for the lymphocytes in two samples, while, for the neutrophils, all three methods showed similar precision. The biggest discrepancy was observed in the case of monocytoid cells, where manual counting via light microscopy showed the lowest CVs in all samples. For the 100-cell differential count with the AI algorithm, the CVs of all samples and all cell categories varied between 3.7% and 92.0%. Meanwhile, the CVs for the 800-cell differential with the AI algorithm, in all cell populations and all three samples, varied between 1.2% and 49.7% and were generally lower, except for the monocytoid cells in one sample. In this sample, the monocytoid cells were the smallest cell population, accounting for between 0.8% and 3.1% of all cells, and the previously described misclassification issue of fragmented recognition was noted.

## 4. Discussion

This study compared automated and manual methods for taking the TNCC and differential cell counts from the CSF of dogs. The results obtained with the Sysmex XN-V highlighted the importance of manual regating, as graphically demonstrated on scattergrams. Without manually set gates, the BF mode often misclassified the PMN cells as debris, while the MN cells were partly counted as MN and PMN cells as well as debris, which could further lead to deceptively low BF-TNCC and BF-WBC counts, as well as inaccurate percentage distributions in the two-part differentiation into BF-PMN% and BF-MN%. For this reason, only the results collected after regating were analyzed further in this study. Before regating, the misclassification issue was mainly observed on scattergrams with moderate to marked pleocytosis. Similar gating errors were previously observed in a similar study that we performed with equine bronchoalveolar lavage samples [28]. However, to our knowledge, no other study has yet explicitly discussed the importance of regating on any hematology instrument. Indeed, very few studies have referred to regating in general: in one study, “gating out” of cellular debris on a Coulter Counter^®^ using vital stain or cell-specific antibodies in mice was mentioned [34], and in another study, this one of bronchoalveolar lavage in animal research for the pharmaceutical industry, a disadvantage of the ADVIA instrument was noted compared to the Sysmex hematology analyzer in that the former has no custom gating settings [35]. Furthermore, the authors also suspect that the observed lack of correlation seen in the Sysmex XN-V study from 2020 with canine CSF could at least be partly explained by incorrect gating since both debris and the well-demarcated cloud of dots in the PMN cell area were of the same color in the depicted scattergrams [25].

After regating, the measurements of the BF-TNCC and the BF-WBC were identical in all samples. For this reason, only the BF-TNCC results were included in our further detailed analysis. Similar results, showing a correlation of r > 0.83 for both the Sysmex BF-WBC and the Sysmex BF-TNCC with the manual TNCC in canine CSF samples with pleocytosis (>5 cells/µL), were observed in the aforementioned study from 2020 [25].

The samples were classified as either unremarkable or having pleocytosis based on the TNCC. In comparison to the manual TNCC, 5.6% more samples were classified as having pleocytosis according to the automated TNCC. Although all of these samples showed borderline pleocytosis, we advise that the two methods should not be used interchangeably when monitoring individual animals.

It must also be mentioned that neither the manual TNCC nor the BF-TNCC can be considered the gold standard for TNCC measurements of the CSF. However, the BF-TNCC is arguably the more accurate of the two since more cells are quantified, suggesting a higher precision.

For the Sysmex XN-V two-part differential cell count, we obtained similar results to what has been reported using the ADVIA 2120 in the CSF assay mode [19]. In contrast, the study from 2020 with the Sysmex XN-V showed no correlation at all for either MN or PMN cells, which was most likely due to the incorrect gating mentioned previously [25]. Nonetheless, the two-part differentiation is limited in its practical applicability. Therefore, in future studies, specific gates should be developed to separately quantify different cell populations. This goal seems to be attainable since three distinct cell populations could be clearly distinguished on some scattergrams with pleocytosis in this study. To date, no studies in veterinary medicine have yet tested the ability of the Sysmex XN-V BF mode to detect eosinophils, but the detection of these cells using the analyzer’s BF mode has been set as a research objective [36], and we propose that establishing a separate gate for the eosinophils should be considered as well.

Furthermore, in this study, all samples with pleocytosis were classified by their type using both the automated and manual two-part differential cell counts. All samples classified as having mixed-cell pleocytosis and most samples classified as having mononuclear pleocytosis according to the manual differential count via light microscopy were also classified in the same pleocytosis category according to the results of the Sysmex BF mode. Meanwhile, the samples being classified as having polymorphonuclear pleocytosis according to the manual differentiation were mostly classified in the category of mixed-cell pleocytosis according to the Sysmex BF mode. It can be argued that the automated method may be more accurate than the manual one as it allows for the differentiation of more cells than the 100-cell differential count performed manually. However, the key takeaway seems to be the importance of using one method rather than mixing the two, since these results demonstrate again that the methods should not be used interchangeably.

Regarding precision, the Sysmex BF mode outperformed the manual method for the TNCC in the case of a low-cell-count sample, as was expected, but showed relatively similar CVs with the manual method in the case of a high-cell-count sample. These results demonstrate the superior performance of the Sysmex BF mode in low-cell-count samples. The linearity for the TNCC was also acceptable.

This study also showed, for the first time, that AI can accurately recognize lymphocytes, neutrophils and monocytoid cells in most cases, when digital images of virtual CSF cytospin preparations are analyzed using a pre-developed algorithm. The lower correlation coefficient of the monocytoid cells can be explained by their pleomorphic cytologic appearance, with, in some cases, morphological features similar to those of lymphocytes or neutrophils [37]. Some difficulty regarding the differentiation between lymphocytes and monocytoid cells was noted even during the manual differentiation of a few samples in this study. While, to the authors’ knowledge, no similar studies of CSF have been performed in veterinary medicine, the results of this study are supported by two publications in human medicine from 2022. The accuracy of an AI algorithm in the recognition of monocytoid cells was noted to be high in those two studies but still lower than for the cell populations of lymphocytes and neutrophils [26,27]. Regarding research on AI cell recognition in veterinary medicine, a study of equine bronchoalveolar lavage showed correlations of between r = 0.85 and 0.92 with a manual method for the identification of alveolar macrophages, lymphocytes, neutrophils and mast cells [28], and a study with bovine uterine cytobrush samples demonstrated adequate agreement between AI and the manual method for >5% and >10% PMN cell thresholds [38].

When comparing the pleocytosis classifications based on the three-part differential counts, as determined using the three different methods, our results varied notably in all categories of pleocytosis. The greatest concordance, of 73.8% and involving all pleocytosis categories, was observed between the differential counts taken manually and by an AI algorithm on digital images. This was not surprising as both methods analyzed the same images of cells, but it underlines the strong performance of the AI algorithm. Unexpectedly, the best agreement was observed for mononuclear pleocytosis. The discrepancies that we observed between the results of these two methods on the same images can mainly be explained by various classification errors for the AI-algorithm-based differential count, as discussed in the next paragraph. Meanwhile, the greatest discordance was observed between the manual differential cell count via light microscopy on the cytospin preparations and both methods applied on the digital images, with only 8.2% of samples concordant for all three methods. The most common mismatch was noted between neutrophilic and lymphocytic pleocytosis. This discordance was surprising and could be explained by an uneven distribution of the cells on the cytospin preparations, the relatively large number of samples with borderline differential counts or the relatively high imprecision when counting only 100 cells [39].

Regarding the classification issues, the following errors appeared to apply to numerous samples: misidentification of several closely located or touching cells as one, fragmented misclassification of one cell as several and misidentification of staining artifacts or unidentified artifacts as various cells. The first two errors were similar to those observed in the study of the AI differential counts for digital images of equine bronchoalveolar lavage samples [28]. A possible solution to the issue of counting several closely packed cells as one cell could be adjusting the cytospin preparation protocol to decrease the cell density on the slide. Such adjustments would be most beneficial for samples with moderate to marked pleocytosis, and they could be researched in future studies. Another notable issue was the misclassification of whole cell populations due to inadequately stained slides. In this case, the cells appeared paler than their counterparts on the training images, meaning the recognition and classification of entire cell populations was severely impaired, emphasizing the tone-sensitive nature of AI algorithms. No staining protocol error was found in these cases, but careful assessment of the staining quality of slides before scanning is advised to allow for restaining if needed. An unexpected discovery was the misclassification of the canine eosinophils as neutrophils, which was explicitly demonstrated in the sample with eosinophilic pleocytosis. No similar issue was previously observed with equine bronchoalveolar samples, which could be explained by the very distinct appearance of equine eosinophils in terms of their prominent intracytoplasmic granules [40], which may prevent any misidentification issues with an AI algorithm, though the same is not necessarily the case for canine eosinophils.

We must highlight one of the main advantages of taking the differential cell count using the AI algorithm, which is the ability to rapidly count several thousand cells without any human bias or assistance and without requiring staff to have technical knowledge of either cell-recognition or information technologies [38]. The precision of AI appears to increase with the number of counted cells, since for the 100-cell differential count, manual differentiation both on digital images and via light microscopy on cytospin preparation slides demonstrated superior results, but for the 800-cell differential count, the AI algorithm showed lower CVs for lymphocytes and neutrophils, as well as monocytoid cells in most cases. Nevertheless, the AI-algorithm-based differentiation has some drawbacks: the equipment is expensive; the development of the neural network algorithm is time-consuming and requires high-quality samples containing all of the cell populations to be classified, and classification issues can lead to various inaccuracies. Nevertheless, the differentiation via an AI algorithm still holds immense potential and seems very promising.

The main limitation of this study was the highly standardized conditions under which the algorithm was developed, which preclude its successful implementation if the visual appearance of the cells is even slightly altered. Likewise, any changes to the sample preparation protocol, including using different stains or altering the staining procedure, may render the developed algorithm inapplicable. This was explicitly demonstrated when a pair of slides were accidentally stained paler than expected, and the algorithm consequently misclassified or failed to identify whole cell populations because the cells appeared of paler color than their counterparts on the slides used for algorithm training had been. Further multicenter studies are needed to broaden the applicability of the algorithm, including studies using different stains and sample preparation protocols. Moreover, additional cell types such as eosinophils, surface epithelial cells and erythrocytes should also be investigated. In addition, the algorithm could be further optimized by emphasizing the cell size as one of the recognition criteria and thus avoiding the misclassification of several closely packed cells as one. In the current study, however, no further optimization of the algorithm could be achieved once the similarity had reached 0.75, as the similarity failed to improve upon increasing the number of training images. Lastly, it must be mentioned that a possible approach to overcoming staining issues altogether is the application of virtual staining, as similar approaches have been successfully used in histopathology with hematoxylin and eosin staining [41,42,43,44].

## 5. Conclusions

This study has demonstrated the applicability of the Sysmex XN-V BF mode for determining the TNCC in canine CSF. However, we were required to generate a manual gate in order to obtain accurate two-part differential cell count measurements. This manual gate could then consequently be applied to all canine samples without the need for individual optimization and should thus be viewed by the manufacturer as an opportunity to improve their settings. An advantage to the automated approach is that the Olympus VS200 software can be operated without specific computer programming skills in order to generate an AI algorithm. Furthermore, the AI three-part differential cell count offers similar precision to the 100-cell differential cell count obtained with manual methods and better precision than the 800-cell differential cell count obtained likewise. In the authors’ opinion, AI algorithms, in general, offer a promising tool for the assessment and differentiation of canine CSF samples. Nonetheless, optimization is required as classification issues still occur. Such optimization may be realized through multicenter studies aiming to broaden the applicability of algorithms to further cell types and sample preparation protocols.

## Figures and Tables

**Table 1:** Overview of the tested methods, compared parameters and the number of samples for each method and parameter.

	Parameters	TNCC	Two-Part Differential Cell Count	Three-Part Differential Cell Count
Methods				
Sysmex XN-V BF mode (automated counting)		161 CSF samples	65 CSF samples with pleocytosis	
Olympus VS200 scanner and software (a) Manual differentiation on digital images (b) Automated differentiation on digital images using AI algorithm				62 CSF scanned cytospins with pleocytosis, digital images of 100 cells each
Light microscopy (manual counting)		161 CSF samples, Fuchs-Rosenthal chamber	65 CSF cytospins with pleocytosis, 100 cells counted	64 CSF cytospins with pleocytosis, 100 cells counted *

AI—artificial intelligence; BF—body fluid; CSF—cerebrospinal fluid; TNCC—total nucleated cell count. * Only for classification of the samples with pleocytosis by the type of pleocytosis.

**Table 2:** Comparison of the TNCC and the two-part differential cell count results from the Sysmex XN-V BF mode with those from the manual method.

Parameter	Passing–Bablok Regression Analysis (95% CI Slope and Intercept)	Bias Bland–Altman Difference Plot (95% CI)	r	*p*-Value
BF-TNCC	y = 0.64 + 1.18x (Slope: 1.14 to 1.27 Intercept: 0.25 to 0.66)	15.19 cells/µL (−11.17 to 41.56)	0.91	<0.001
BF-WBCs	y = 0.64 + 1.18x (Slope: 1.14 to 1.27 Intercept: 0.25 to 0.66)	15.19 cells/µL (−11.17 to 41.56)	0.91	<0.001
BF-MN%	y = 11.67 + 0.88x (Slope: 0.77 to 0.97 Intercept: 2.99 to 23.08)	4.95% (1.34 to 8.56)	0.89	0.03
BF-PMN%	y = 0 + 0.88x (Slope: 0.77 to 0.96 Intercept: 0 to 1.69)	−4.95% (−8.56 to −1.34)	0.89	0.03

BF—body fluid; CI—confidence interval; MN—mononuclear; PMN—polymorphonuclear; *p*-value—Wilcoxon signed-rank test; r—Spearman’s rank correlation coefficient; TNCC—total nucleated cell count; WBCs—white blood cells.

**Table 3:** Classification of the samples by the TNCC as either unremarkable or having pleocytosis.

Parameter	Number of Unremarkable Samples (TNCC ≤ 5 Cells/µL)	Number of Abnormal Samples (TNCC > 5 Cells/µL)
BF-TNCC	96	65
BF-WBCs	96	65
Manual TNCC	105	56

BF—body fluid; TNCC—total nucleated cell count; WBC—white blood cells.

**Table 4:** Classification of the samples with pleocytosis by the two-part differential count according to the type of pleocytosis.

Method	Number of Samples with Mononuclear Pleocytosis ^1^	Number of Samples with Mixed-Cell Pleocytosis ^2^	Number of Samples with Polymorphonuclear Pleocytosis ^3^
Sysmex BF differential count	39	18	8
Manual differential count via light microscopy	39	6	20

BF—body fluid; ^1^ ≥70 MN%; ^2^ both MN% and PMN% < 70; ^3^ ≥70 PMN%.

**Table 5:** Classification of the samples with pleocytosis by the three-part 100-cell differential count according to the type of pleocytosis.

Method	Number of Samples with Lymphocytic Pleocytosis ^1^	Number of Samples with Mononuclear Pleocytosis ^2^	Number of Samples with Mixed-Cell Pleocytpsis ^3^	Number of Samples with Neutrophilic Pleocytosis ^4^	Number of Samples with Eosinophilic Pleocytosis ^5^
AI differential count on digital images	15	10	20	17	0
Manual differential count on digital images	18	7	10	26	1
Manual differential count via light microscopy	26	12	4	19	1

^1^ ≥70% cells are lymphocytes, ^2^ ≥70% cells are mononuclear, but <70% cells are lymphocytes, ^3^ all cell types are <70% and mononuclear pleocytosis does not apply, ^4^ ≥70% cells are neutrophils, ^5^ ≥20% cells are eosinophils [5].

**Table 6:** Comparison of precision for the TNCC between the automated Sysmex BF TNCC and the manual counting with a hemocytometer.

Method	Sample Cellularity	Mean TNCC (Cells × 10^6^/µL)	n	SD (Cells × 10^6^/µL)	CV (%)
Sysmex BF	Low	2.4	10	0.5	21.5
	High	757.0	6	36.5	4.8
Manual	Low	1.5	8	0.7	47.5
	High	127.3	6	4.9	3.8

BF—body fluid; CV—coefficient of variation; n—number of replicates; SD—standard deviation; TNCC—total nucleated cell count.

**Table 7:** The precision of the three-part 100-cell differential count as determined by three methods (applying AI algorithm and manual counting on digital images and via light microscopy on cytospin preparations), as well as the three-part 800-cell differential with the AI algorithm.

			Monocytoid Cells %				Lymphocytes %				Neutrophils %			
			Manually Microscope 100 Cells	Manually Image 100 Cells	AI Image 100 Cells	AI Image 800 Cells	Manually Microscope 100 Cells	Manually Image 100 Cells	AI Image 100 Cells	AI Image 800 Cells	Manually Microscope 100 Cells	Manually Image 100 Cells	AI Image 100 Cells	AI Image 800 Cells
Sample No. (TNCC cells × 10^6^/µL)	1 (337 cells × 10^6^/µL)	Average	13.3	10.2	10.7	11.9	3.6	3.1	3.7	5.1	83.1	86.6	85.5	83.0
		CV%	29.3	42.6	40.5	7.5	80.7	70.8	75.8	13.7	4.6	6.3	7.6	1.2
	2 (1170 cells × 10^6^/µL)	Average	10.1	5.5	3.0	2.4	4.3	1.5	7.0	10.1	85.6	92.7	90.0	87.5
		CV%	21.4	40.5	92.0	49.7	72.0	87.4	38.5	10.7	3.1	2.8	5.1	2.1
	3 (1451 cells × 10^6^/µL)	Average	5.5	3.6	5.3	4.4	1.9	1.1	3.7	3.7	92.6	95.3	91.0	91.9
		CV%	37.6	61.6	51.6	20.1	66.5	75.6	45.6	33.1	2.4	2.9	3.7	2.2

AI—artificial intelligence; CV—coefficient of variation.

## Data Availability

Dataset available on request from the authors.

## Supplementary Materials

The following supporting information can be downloaded at: https://www.mdpi.com/article/10.3390/ani14111655/s1, Table S1: Raw data for TNCC, Table S2: Raw data for two-part differential cell count, Table S3: Raw data for three-part differential cell count.
